# Assessing Seasonal and Inter-Annual Variations of Lake Surface Areas in Mongolia during 2000-2011 Using Minimum Composite MODIS NDVI

**DOI:** 10.1371/journal.pone.0151395

**Published:** 2016-03-23

**Authors:** Sinkyu Kang, Suk Young Hong

**Affiliations:** 1 Department of Environmental Science, Kangwon National University, Chuncheon 200–701, Republic of Korea; 2 Department of Agricultural Environment, National Academy of Agricultural Science, Wanju 565–851, Republic of Korea; University California Los Angeles, UNITED STATES

## Abstract

A minimum composite method was applied to produce a 15-day interval normalized difference vegetation index (NDVI) dataset from Moderate Resolution Imaging Spectroradiometer (MODIS) daily 250 m reflectance in the red and near-infrared bands. This dataset was applied to determine lake surface areas in Mongolia. A total of 73 lakes greater than 6.25 km^2^in area were selected, and 28 of these lakes were used to evaluate detection errors. The minimum composite NDVI showed a better detection performance on lake water pixels than did the official MODIS 16-day 250 m NDVI based on a maximum composite method. The overall lake area detection performance based on the 15-day minimum composite NDVI showed -2.5% error relative to the Landsat-derived lake area for the 28 evaluated lakes. The errors increased with increases in the perimeter-to-area ratio but decreased with lake size over 10 km^2^. The lake area decreased by -9.3% at an annual rate of -53.7 km^2^ yr^-1^ during 2000 to 2011 for the 73 lakes. However, considerable spatial variations, such as slight-to-moderate lake area reductions in semi-arid regions and rapid lake area reductions in arid regions, were also detected. This study demonstrated applicability of MODIS 250 m reflectance data for biweekly monitoring of lake area change and diagnosed considerable lake area reduction and its spatial variability in arid and semi-arid regions of Mongolia. Future studies are required for explaining reasons of lake area changes and their spatial variability.

## Introduction

Mongolia possesses abundant inland water resources, although large numbers of rivers, lakes and wells have disappeared or were depleted between the late 1990s and early 2000s [1, 2]. These water resource reductions were closely linked with an exacerbation of desertification and deterioration of local socio-economic systems in arid and semi-arid regions of Mongolia [3]. In addition to reported temperature increases of 1.6°C since the 1960s [4], the annual precipitation substantially decreased during early 2000s, which may have altered the regional water balance of grassland ecosystems to reduce the available water resources in Mongolia. When combined with climate change or variability, the regional water balance is complicated by the presence of glacier and permafrost areas located in the Altai mountains [5] and north-central high latitude regions [6], respectively. Hence, it is expected that regional water balance processes are spatially and temporally heterogeneous across different climatic and geographic regions in Mongolia.

Reliable monitoring of temporal water resource dynamics is important for sustainable management of dryland areas. Lake water is the largest water resource (approximately 500 km^3^), providing 80% of the total freshwater supply in Mongolia [1]. In addition to supplying local water demands, lake water is an important indicator of temporal variations of basin-scale groundwater resources [7], which are commonly used by nomadic people in rural Mongolia [8]. Hence, rapid and reliable monitoring of lake water resource over wide geographic areas may provide useful indirect estimates of basin-scale groundwater changes in arid and semi-arid regions.

Satellite remote sensing data have been applied for monitoring lake water resources and lake surface area specifically. Multi-temporal satellite remote-sensing images provide useful tools for monitoring temporal changes in lake area. For example, Landsat images were used to investigate dynamic changes in playa lakes in the Monegros Desert in Spain [9]. Tao et al. (2015) [2] surveyed extensive Landsat images from the 1970s to 2000s, and reported rapid loss of lakes on the Mongolian Plateau due to reduction of precipitation in Mongolia and mining and cultivation activities in Inner Mongolia, respectively. Various types of satellite data from Landsat, ASTER, MODIS, AVHRR, and JERS-1 were applied to analyze long-term changes in lake surface areas in central Asia [10]. RADASAT SAR images were used to investigate Orog Lake in southern Mongolia [11]. In previous studies, high spatial resolution images have been widely preferred for lake area detection, whereas the application of coarse-resolution data, such as Moderate Resolution Imaging Spectroradiometer (MODIS) and Advanced Very High Resolution Radiometer (AVHRR) data, has been limited to large lakes or reservoirs [10, 12–15].

In arid and semi-arid regions, closed lakes are common and show extensive gradients [2] and considerable seasonal fluctuations in lake areas [10]. Either reduction trend of lake size or lake depletion can be a prominent symptom of exacerbation of region-scale drought hazard. The seasonal high variability of lake areas in arid and semi-arid regions needs fine temporal-scale monitoring, from which the long-term trend or seasonal depletion of lake areas can be captured. For this, high temporal resolution satellite data such as MODIS are preferred, although the coarse spatial resolution of MODIS may increase the uncertainty of area detection for small lakes. The aforementioned tradeoff of using MODIS data has not been fully investigated, especially for regions with considerable lake area gradients and seasonal fluctuations, such as the arid and semi-arid regions of Mongolia.

This study aimed to develop a monitoring method to detect seasonal (i.e. weekly or monthly) lake surface areas using 250m MODIS-derived spectral indices. The non-frozen season lake areas were determined for 73 lakes across Mongolia from 2000 to 2011 at a 15-day interval. The reliability of lake area detection from 250 m MODIS dataset was evaluated based on the lake area derived from Landsat TM and ETM+ images at 30 m resolution. The MODIS-derived seasonal lake area datasets were applied to examine inter-annual trend of lake surface areas.

## Materials and Methods

### Study areas and data collection

**Fig 1:**
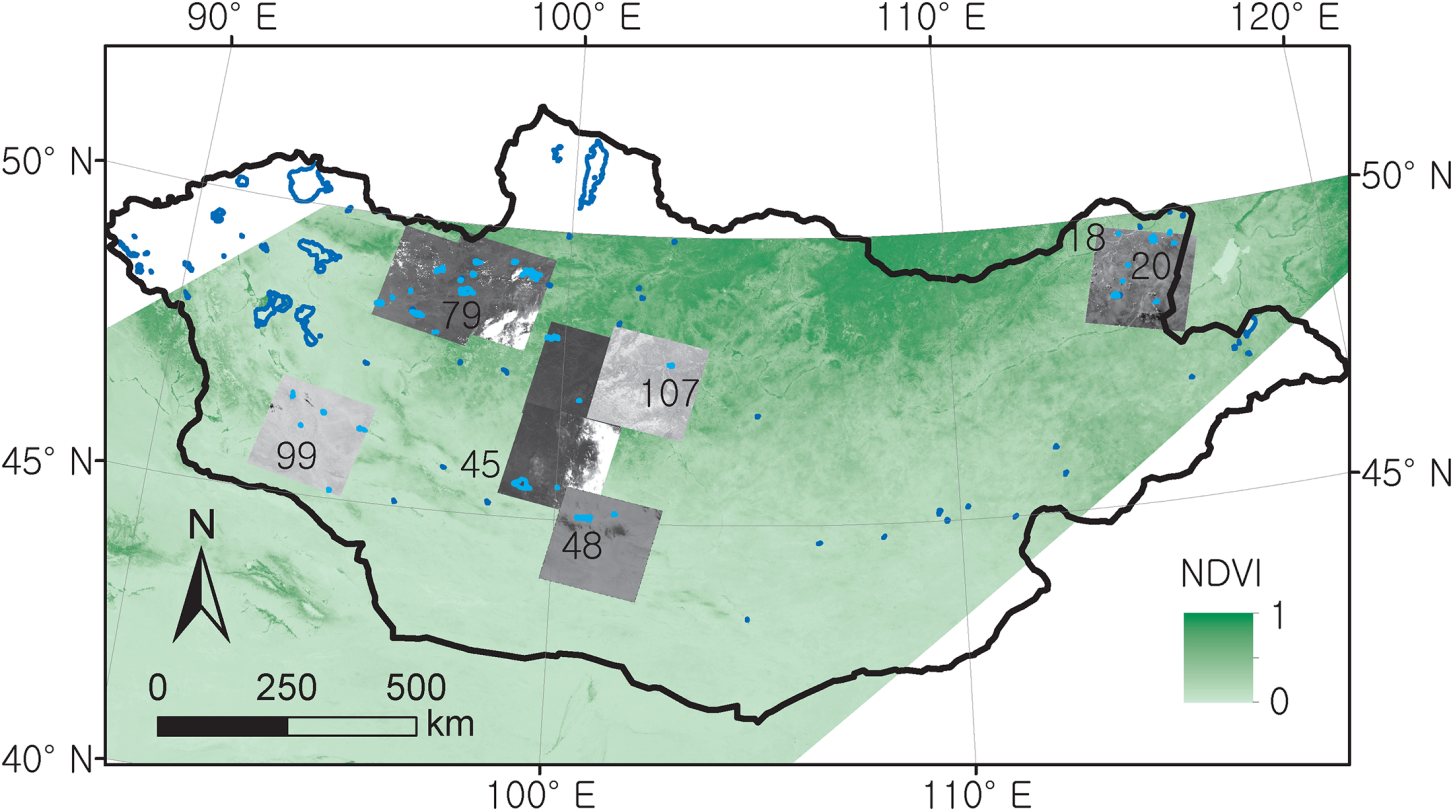
A sample MODIS 250m NDVI overlaid with a GIS lake data layer (red polygons) and Landsat images. Blue polygons are lakes with areas greater than 6.25 km^2^ in Mongolia. Light blue polygons on Landsat images are the 28 evaluation lakes. Data sources are USGS LPDAAC for MODIS NDVI; USGS GLOVIS for Landsat images; Digital Chart of the World for lake polygons, respectively.

Lakes with areas greater than 6.25 km^2^ were selected to test our MODIS-based lake area detection method and to examine seasonal and inter-annual lake area variations from 2000 to 2011 (Fig 1). The threshold lake area of 6.25 km^2^ corresponds to approximately 100 MODIS pixels at 250 m resolution. By applying this threshold area, our study focuses only on lakes that are large enough to present area changes from dry to wet years but remain small enough to examine water detection problems based on lake-boundary pixels. The lake selection was based on GIS lake polygon data from by the Ministry of Environment and Green Development of Mongolia, which includes a total of 109 lakes greater than 6.25 km^2^ in Mongolia. However, this study targets only 73 lakes located within two MODIS Land tiles (tile IDs, H24V04 and H25V04; Fig 1). For 28 of these lakes, the MODIS-derived lake areas were compared with the lake areas detected based on Landsat TM and ETM+ images at a pixel resolution of 30 m. For the 28 evaluation lakes, the MODIS-derived lake areas were compared with the lake areas detected based on the Landsat image. The evaluation lakes showed clear gradients in lake area from 6.4 to 201.5 km^2^ across different geographic regions of Mongolia (Fig 1).

Multiple datasets were collected for a period from 2000 to 2011, which included Terra MODIS level-5 daily 250 m land surface reflectance (MOD09GQ) [16] and 16-day 250 m NDVI (MOD13Q1) (Land Processes Distributed Active Archive Center, USGS) [17], Landsat TM and ETM+ images (GLOVIS, Earth Resources Observation ad Science Center, USGS), and Tropical Rainfall Measuring Mission (TRMM) monthly 25 km precipitation data (Goddard Distributed Active Archive Center, NASA) [18]. Inland water GIS data and 1-km digital elevation model (DEM) for Mongolia was freely available from Digital Chart of the World through DIVA-GIS website (www.diva-gis.org/Data) and USGS HYDRO1K dataset (lta.cr.usgs.gov/HYDRO1K), respectively. Lakes were extracted from the inland data and then, used for the selection of lakes greater than 6.25 km^2^ in area. All MODIS and Landsat images were projected on a UTM 48N coordinate system.

### Detection and evaluation of lake surface areas

A single near-infrared (NIR) band reflectance, and reflective ratio indices between the NIR and a visible band, such as the Normalized Difference Vegetation Index (NDVI), and the Normalized Difference Water Index (NDWI) are widely used for water classification [2, 13, 19, 20]. The simplest approach of water delineation is to generate a histogram from which water pixels are separated from their background with optimal threshold value [21, 22]. In this study, we choose the threshold-based water classification method and the MODIS-derived 250 m NDVI to evaluate MODIS applicability for reliable monitoring of seasonal lake areas across lake-size gradients in Mongolia. It is because the NIR threshold is less robust for high concentration of water-body suspended matter and atmospheric effect [23] and the NDWI requires green band reflectance available only at 500m spatial resolution.

Generally, water has negative NDVI [13] but the mineral or chlorophyll contents of water or seasonal sun-earth-sensor geometry may increase the NDVI up to near-zero positive values [22]. Mixed land-water or shoreline vegetation within lake-boundary pixels can occasionally result in large NDVI values [24]. In addition, frequent Asian Dust passing over a lake water body can result in erroneously high NDVI values due to higher the infrared reflectance of the dust materials relative to the red reflectance. Under these circumstances, the maximum composite method applied for official MODIS 250 m NDVI (MOD13Q1) [17] may fail to detect lake water pixels based on the negative NDVI criteria. The 16-day MOD13Q1 NDVI takes the maximum daily NDVI for the consecutive 16 days.

In this study, minimum composite NDVI was proposed as a novel tool for reducing uncertainty in water classification caused by the occasional short-term high NDVI due to dust materials in arid and semi-arid regions. The minimum composite NDVI is the reverse case of the maximum composite applied to MOD13Q1 NDVI, in which the lowest daily NDVI for a certain period is adopted. Minimum composite NDVI was calculated from daily red (ρ_red_) and infrared (ρ_nir_) reflectances that were extracted from MOD09GQ raw data files together with the corresponding quality control (QC) flags. MOD09GQ provides atmospherically-corrected 250-m surface reflectance of MODIS red and infrared bands [16]. The QC flags provide data quality information, such as the types of cloud contamination. In this study, any reflectance data with cloud contamination were excluded from subsequent processes, and the cloud-free red and infrared reflectance was then used to calculate the daily NDVI (Eq 1).

$$\text{NDVI}=\;\frac{\rho_{\text{nir}}-{\;\rho }_{\text{red}}}{\rho_{\text{nir}}+{\;\rho }_{\text{red}}}$$ (1)

For every 15days, the minimum NDVI was determined as the lowest NDVI from the screened daily NDVI, which is designated hereafter as the 15-day minimum composite NDVI (NDVI_min15_). The above process was implemented for all image pixels and for each year from 2000 to 2011. The 15-day composite was chosen because the 15-day interval was the shorter limit of the NDVI timeseries showing smooth seasonal NDVI variations that were less affected by cloud contamination. The MODIS image procession was done by using IDL (version 8.0, ITT Visual Information Solutions).

In our preliminary tests when the negative NDVI threshold was applied to the original NDVI_min15_, it showed a serious problem regarding the misidentification of certain land pixels as water. This error occurred because the minimum composite method resulted in many negative-NDVI land pixels, although we screened for cloud-contaminated NDVIs using the QC flags of MOD09GQ. The negative NDVI of some land pixels might be due to incomplete information on cloud contamination or other sources of data quality degradation. To resolve this matter, an additional screening step was applied to the original NDVI_min15_. It was assumed that although the land generally has positive daily NDVI values, it can occasionally, but not frequently, have negative daily NDVIs due to data quality degradation of band reflectance. Conversely, it was assumed that water more frequently shows negative daily NDVIs during the 15-day compositing interval. This might be a stochastic effect determined by the quality of the raw MOD09GQ data as well as erratic events, such as the occurrence of Asian Dust, which would be difficult to resolve mechanistically.

The additional empirical screening process was introduced by assuming that water pixels should have a greater number of frequent negative NDVI days than a certain minimum threshold frequency during the 15-day compositing period. By increasing the threshold frequency of negative NDVI days, the misclassified land pixels were correctly identified as land pixels; however, water pixels near lake boundaries were misclassified as land pixels with increasing frequency. The five days of negative daily NDVIs composite was determined as the best threshold frequency criterion for the 15-day interval NDVI, based on both visual interpretations for each lake evaluation and error statistics for all evaluation lakes. This additional screening criterion allowed for the exclusion of the majority of negative-NDVI land pixels from the water detection process.

This study simply applied the negative composite NDVI as the water-classification threshold for both the maximum and minimum composite NDVI datasets. The constant NDVI threshold may cause seasonal over- and underestimation of lake areas due to varying sun-earth-sensor geometry [22]. Nevertheless, it provides a simple objective way to examine both seasonal and inter-annual lake area change because the seasonal errors due to sensor geometry are systematic and oligotrophic lakes are common in Mongolia. The number of water pixels for each lake was counted within a prescribed rectangular region containing the lake. The rectangular region was manually determined for each lake on the lake polygon GIS layer (ArcMap version 9.3, ESRI) by slightly over-fitting to the lake size. Finally, the lake area was calculated by multiplying by the MODIS pixel area (0.0625 km^2^).

Lake water pixels for the 28 evaluation lakes (Fig 1) were identified from Landsat TM and ETM+ images and utilized for evaluating the accuracy of MODIS-based lake area detection. More than one hundred Landsat scenes in total were downloaded, and only selected scenes where target lakes were clearly visible without cloud cover were utilized to estimate lake surface areas. Non-winter images (i.e. June-to-September) were collected to avoid effects of snow or ice cover on the measurement of lake surface area in Mongolia [25]. We conducted image-to-image geometric corrections based on the latest ETM+ image for each region. Atmospheric correction was not applied in this study because it was challenging to obtain atmospheric-profile information for model-based atmospheric correction and also not feasible to find dark or pseudo-invariant feature (PIF) object for empirical or relative atmospheric corrections [26, 27], respectively. This issue might be, however, less problematic in our study because atmospheric correction has little impact on single image-based classification errors when sky is clear and homogeneous [28, 29]. The ISODATA unsupervised classification method [30] using ENVI software (version 4.7, ITT Visual Information Solutions) was applied to identify water pixels. Because the classification results from 7 to 10 classes showed the best fit with the visually recognizable water surface, we applied 10 classes for the ISODATA unsupervised classification for every Landsat image. The classified images contained several water classes that potentially correspond to various lake color conditions, such as clean and dark, shallow and turbid. For each evaluation lake, the water classes were identified by comparing with the visual lake inspection and were then merged as a single water class. In this study, the unsupervised classification process was assisted by visual inspection of surface waters from a Landsat 4-5-3 RGB composition dataset [9, 26, 31].

In summary of data preparation and analysis, the three alternative lake area datasets were produced from MOD13Q1, MOD09GQ, and Landsat images. The former two cases are based on water pixel detection from the maximum and minimum composite NDVIs, respectively, with the water classification criteria of negative composite NDVI. An unsupervised landcover classification method is applied for the Landsat image. In this study, the Landsat-derived lake area was assumed to have the highest quality of lake area detection and therefore was used to evaluate the other two datasets based on MODIS NDVI. The errors were investigated in relations with size and morphology (i.e. perimeter-area ratio) of the 28 evaluation lakes. We analyzed whether the Mongolian lakes showed significant increasing or decreasing inter-annual trends of lake area change between 2000 and 2011 by using the Pearson correlation coefficient. The trend analysis results were then compared with the trend map of annual precipitation change from TRMM dataset.

## Results and Discussion

### Evaluation of MODIS-based lake area detection

**Fig 2:**
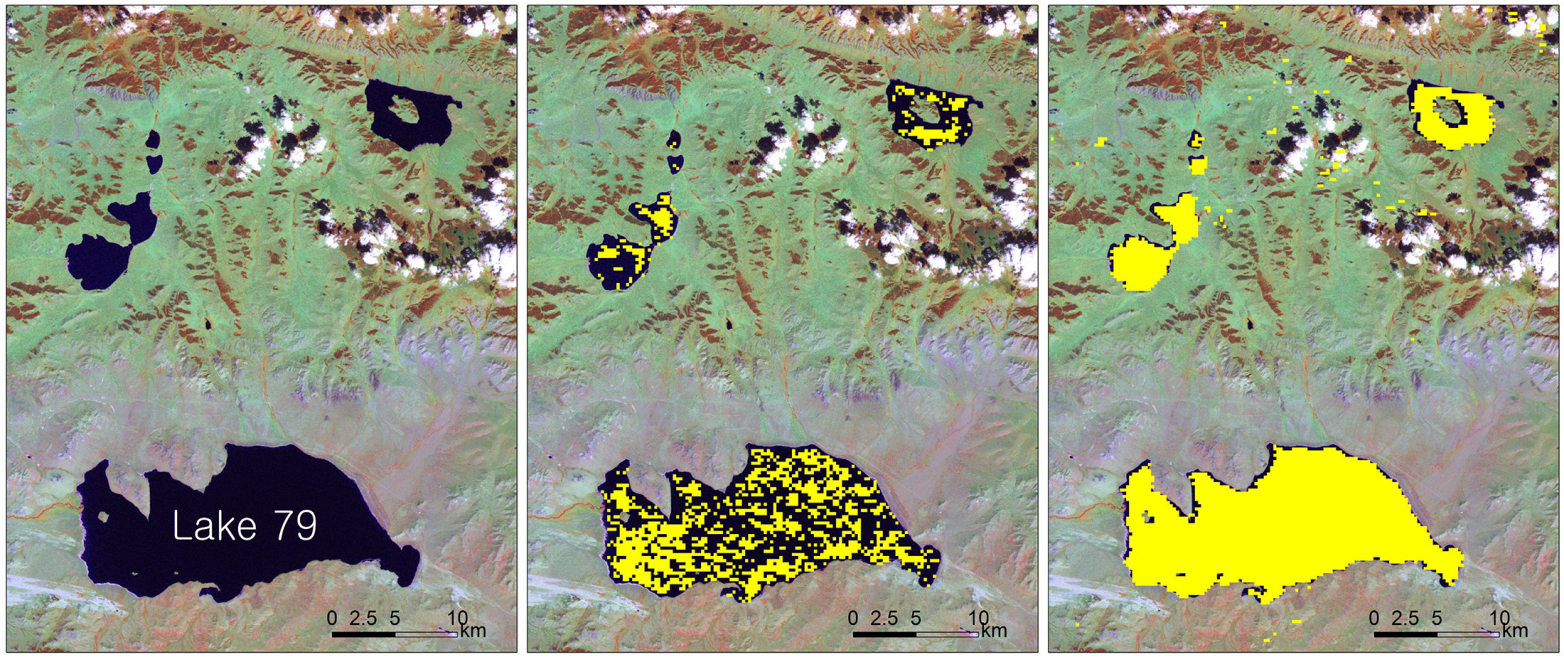
Sample images of lake area detection for the Lake 79 in Fig 1. (a) Landsat TM 4-5-3 RGB composite (2001.229DOY) and water pixels (yellow) from (b) MOD13Q1 16-day Maximum Composite NDVI (2001.225-240DOY) and (c) MOD09GQ 15-day Minimum Composite NDVI (2001.227-241DOY). Data source of Landsat image is USGS GLOVIS.

Lake areas detected based on MOD13Q1 NDVI and NDVI_min15_ were compared with the lake areas from Landsat images. Fig 2 provides an illustration of these comparisons. It was found that MOD13Q1 NDVI consistently underestimated the lake areas for all comparisons, as shown in Fig 2b. Many lake water pixels were not detected based on the detection criteria of negative NDVI. For those pixels, MOD13Q1 NDVI generally showed small positive values less than 0.1 but occasionally had values greater than 0.5. NDVI_min15_ produced a better detection of lake water pixels than did MOD13Q1 NDVI (Fig 2c). An example of lake water detection using NDVI_min15_ and the 5-day negative NDVI threshold is presented in Fig 2c. The detected lake boundary was distinct, and small in-lake islands were also successfully identified.

**Fig 3:**
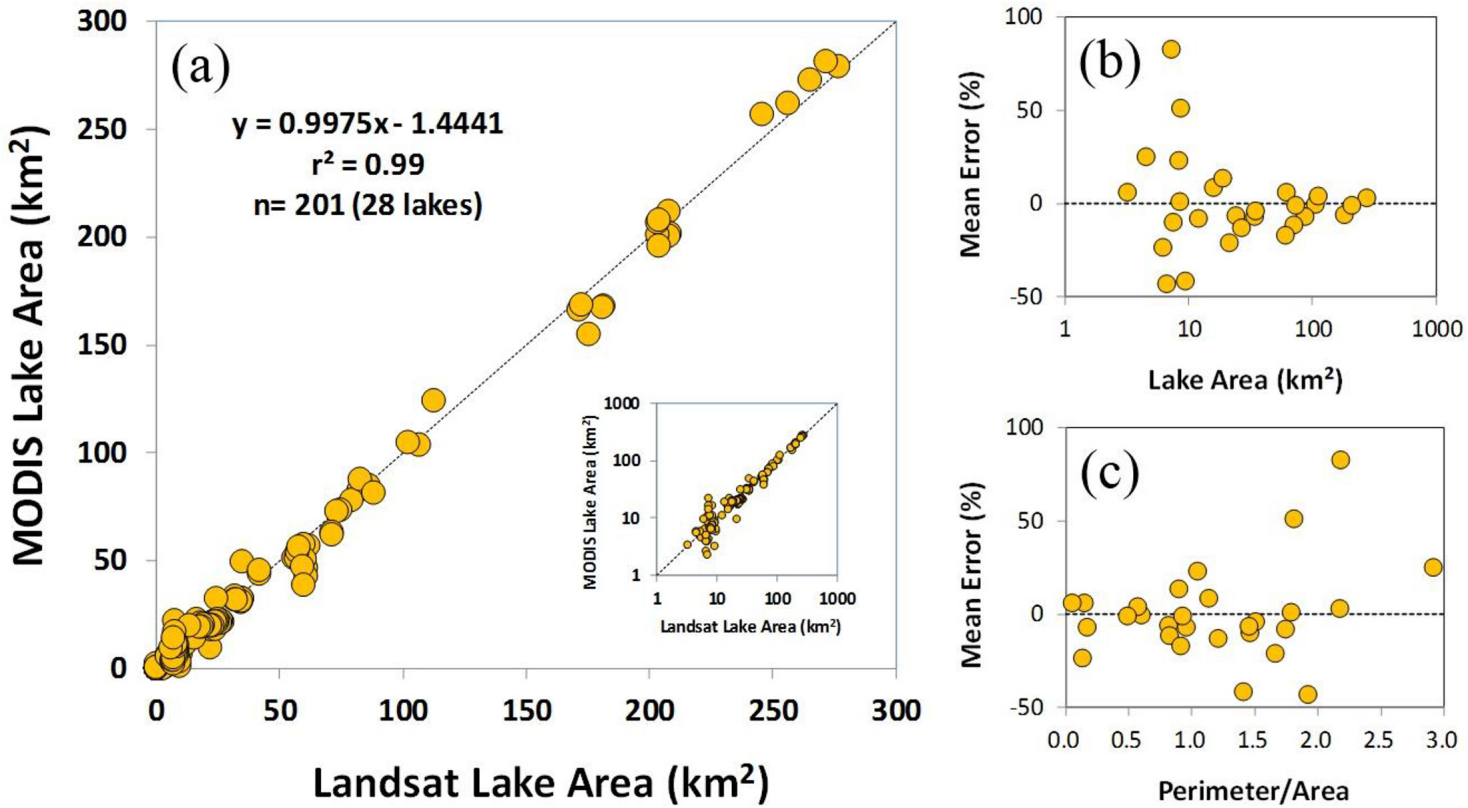
Evaluation of lake area detection. (a) comparison between MODIS-derived and Landsat-derived lake surface areas (km^2^) with a log-scale inlaid figure and errors (%) with respect to (b) lake area and (c) perimeter/area.

The aforementioned detection method for lake water pixels (i.e. the 5-day negative NDVI threshold for NDVI_min15_) was statistically evaluated using Landsat-derived lake areas. For the evaluation lakes (Fig 1), the MODIS-derived lake area explained 99% of the spatial and temporal lake area variations detected based on Landsat images with mean and mean absolute errors of -0.1% and 16%, respectively (Fig 3a). The linear correlation was very high for large lakes but lower for small lakes with areas of approximately 10 km^2^ or less. For the small lakes, the MODIS-derived lake area overestimated the Landsat-derived lake area by +7% with a mean absolute error of 31%, which was contrasted with underestimation (-4%) and smaller absolute error (8%) for the large lakes (Fig 3b). Higher perimeter-to-area ratios were also related with higher errors in MODIS-derived lake area (Fig 3c). For example, the mean and mean absolute errors of lakes with the perimeter-to-area ratios greater than 1.0 were +8% and 23%, respectively, which were compared with respective errors of -3% and 8% for the lower perimeter-to-area lakes.

**Fig 4:**
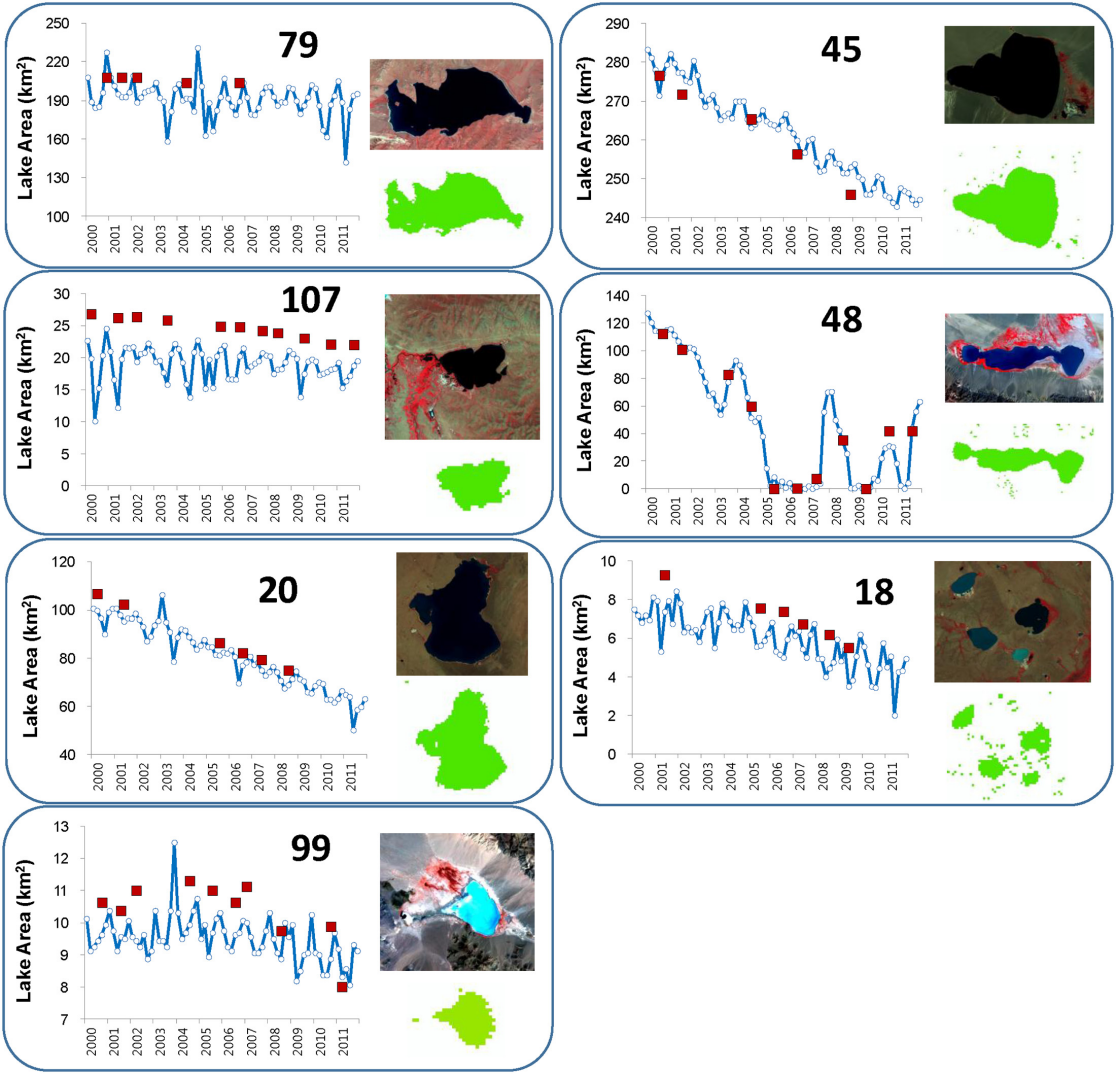
Lake area (km^2^) timeseries from 2000 to 2011. Filled squares and open circles indicate lake areas from Landsat TM/ETM+ and MODIS09GQ, respectively. Upper and lower keymaps show lake water pixels from Landsat and MODIS, respectively. Numbers at the top of each figure are the lake ID numbers shown in Fig 1.

Fig 4 illustrates examples of seasonal and inter-annual variations in lake areas from Landsat and MODIS NDVI_min15_ for the seven lakes numbered in Fig 1. Overall, the lakes showed a maximum size in the early 2000s and then gradually decreased, although with some considerable fluctuations (e.g., Lake 48). MODIS-derived lake areas agreed well with the inter-annual lake area variations. For each lake, greater than 80% of lake area variations were explained by the MODIS-derived lake area, with the exception of lakes 107 and 79. The MODIS-derived lake area underestimated the Landsat-derived lake area with mean errors ranging from -27.0% (lake 18) to -4.0% (lake 79), with the exception of lake 45 (+0.7%). For the seven illustrated lakes in Fig 4, it was found that smaller lake size corresponded with greater mean error (r = -0.79, p < 0.01).

### Seasonal and inter-annual lake area variations

**Fig 5:**
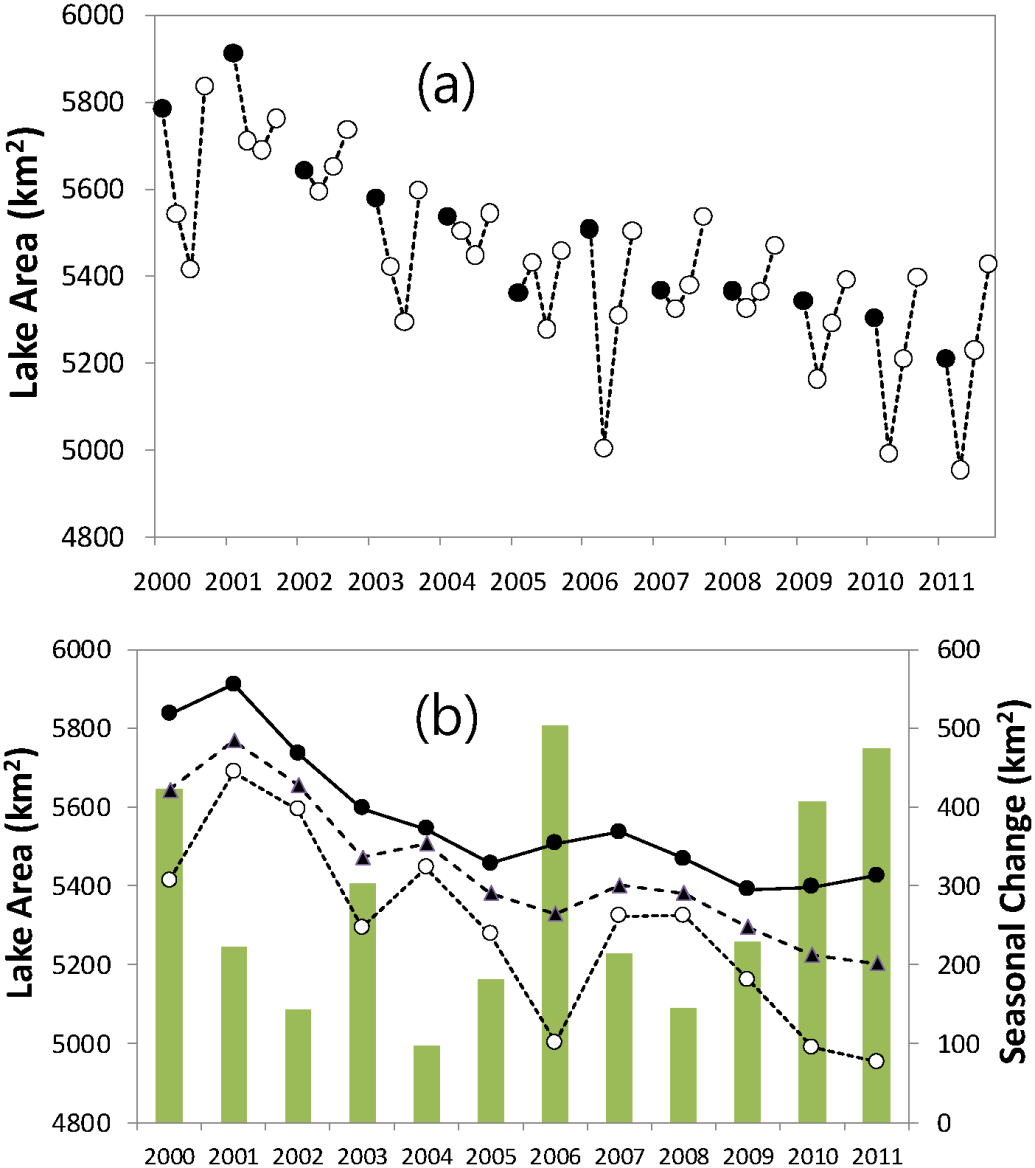
Seasonal (June-to-September) and inter-annual variations of total lake surface areas (km^2^) detected based on MODIS minimum composite NDVI from 2000 to 2011. (a) monthly lake surface area, (b) annual maximum (closed circle), minimum (open circle), and mean (triangle) lake areas with annual range (vertical bar) of seasonal lake area change. Lake areas are shown only for June to September. Closed circle in (a) indicate the lake area in June of each year.

The total lake area of the 73 number of lakes investigated in this study showed considerable seasonal and inter-annual variations. The total lake area decreased by -9.3% at an annual rate of -53.7 km^2^ yr^-1^ from 2000 to 2011 (r^2^ = 0.86, p < 0.01; Fig 5). Among the 73 lakes, nine lakes larger than 100 km^2^ explained 40% of the total lake area reduction, while 7 medium (50–100 km^2^) and 57 small-sized (< 50 km^2^) lakes accounted for 30% reduction each, respectively. The annual reduction rates were statistically higher (-16.2 km^2^ yr^-1^, p < 0.01) for lakes over 100 km^2^ than those of small (-12.8 km^2^ yr^-1^) and medium-sized (-11.3 km^2^ yr^-1^) lakes. In spite of the highest annual reduction rate, due to its large mean lake area (343.6 km^2^), the surface area of large-sized lakes decreased only by 5.2% between 2000 and 2011, while the medium (-25.4%) and small-sized (-22.6%) lakes showed relatively larger reductions.

Seasonal drying and recharging phases are distinct. For every year, the minimum lake area was detected in July or August, whereas the maximum area occurred in June or September. The minimum lake area showed greater reduction rate with considerable inter-annual variation (-52.0 km^2^ yr^-1^ and r^2^ = 0.79, p < 0.001) than the maximum lake area (-42.5 and 0.65, p < 0.001).

**Fig 6:**
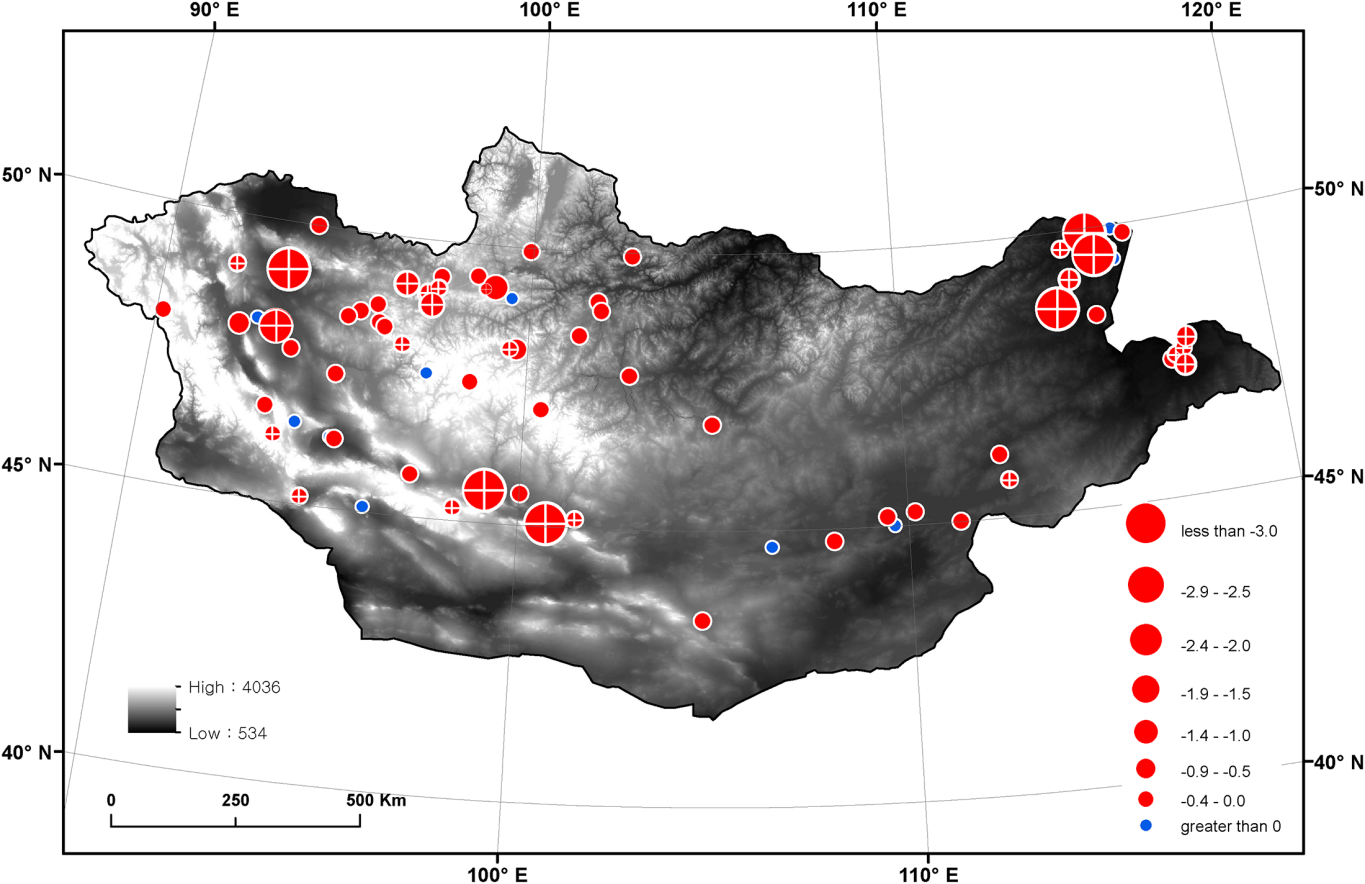
Annual rate of lake area change (km^2^ yr^-1^) between 2000 and 2011. Red and blue circles indicate decreasing and increasing trends, respectively. The circle sizes are adjusted based on the absolute rate of change. The cross check circle indicates a significant trend of lake area change at the p < 0.05. The background is a digital elevation map (DEM, m) from the USGS HYDRO1K DEM data.

Individual lakes showed considerable spatial variation in the inter-annual seasonal lake area changes (Fig 6). The annual rates of seasonal lake area change ranged from -8.9 km^2^ yr^-1^ to +0.22 km^2^ yr^-1^. Generally, lakes showing a higher reduction rate were distributed in the western, southern, and eastern parts of Mongolia, whereas north-central and south-western lakes showed a lower reduction rate and, in certain cases, an increasing rate. Among the 73 studied lakes, significant decreasing trends in the annual mean lake area (cross check circles in Fig 6; p < 0.05) were found only at 28 lakes where mostly appeared collectively in several regions: cool-and-dry western valley lakes, southern big Gobi lakes, eastern lowland lakes, and north-western highland lakes. Those collective patterns imply presence of certain regional hydrology resulting in lake area reduction. Although 12 lakes showed a trend of increasing lake area, this was not statistically significant (p > 0.05). Across the lakes, the annual rate of lake area change appeared strongly correlated with lake area (e.g. in comparison with year-2000 lake area, Pearson correlation coefficient, r = -0.44, p < 0.01). The lakes with significant trends showed, in average, steeper annual rate (mean and standard deviation, -1.4±2.1 km^2^ yr^-1^) and larger lake area (148.2±348.5 km^2^) than those of the other lakes (-1.1±0.3 km^2^ yr^-1^ and 31.1±137.8 km^2^), respectively (t-test, p < 0.05).

## Discussion

Multiple satellite sensors enable researchers to monitor lake water level and area change that facilitate estimation of lake volume change [32]. Satellite altimetry provides reliable measures at the lake level but is generally confined for lakes large enough (ca. over 100 km^2^ in width) to detect or easily accessible [32, 33]. Whereas, because of its extensive applicability, satellite-based lake area monitoring using various optical and thermal remote sensing data has been implemented widely and provides reliable measurement on regional lake hydrology in arid and semi-arid regions. In this study, seasonal and annual lake area variations for 73 lakes in Mongolia were monitored with MODIS 250-m reflectance data from 2000 to 2011. Our lake detection method using the 15-day minimum composite NDVI enabled biweekly monitoring of lake area change with a reliable detection performance of -2.5% error relative to the Landsat-derived lake area values for the 28 evaluation lakes. The errors increased with increasing perimeter-to-area ratios but decreased with lake size, which requires further development of an enhanced detection method for lakes smaller than approximately 10 km^2^. An additional test on the NDWI applicability is also necessary to evaluate compensation between the coarse spatial resolution (500 m) and high water detection ability [19, 20].

Our satellite-based lake area monitoring indicates remarkable lake area reductions in Mongolia. This study reported -9.3% decrease between 2000 and 2011 with an annual rate of -53.7 km^2^ yr^-1^ for this period. Tao et al. (2015) [2] also reported lake area reduction (-2.4%) in Mongolia between the late 1980s and 2010. They showed slight increase for lakes over 50 km^2^ but substantial reductions (-27.9% and -19.7%) for lakes with an area of 1–10 km^2^ and 10–50 km^2^, respectively. This study evaluated greater lake area reductions in the western, southern, and eastern regions of Mongolia, while the north-central lakes showed slight-to-moderate reduction rates and even increasing rates for some lakes.

Country-wide lake area reduction in Mongolia found in this study may be relevant with recent hot-and-dry climate desiccating land surface water [25]. It was reported dry 2000s following wet 1990s and rapid warming since 1980s, both of which imply hot-and-dry climate regime in Mongolia during 2000s. It seems, however, the considerable spatial variability in lake area change (Fig 6) requires further evaluations on regional characteristics of climate and hydrological processes. Changes in lake area are the result of changes in the basin-scale water budget, including rainfall, snowmelt, evapotranspiration, river discharge, and anthropogenic water use [25]. Different lake morphologies (e.g., size and depth) can also result in different lake area changes for a given change in water budget.

Though this study did not address such factors explicitly, it is worthy of noting that two speculations. First, even during the dry 2000s, TRMM-based annual precipitation trend showed the increasing trend in the north-central region (data not shown). Second, the southern fringe of the Siberian permafrost zones vulnerable to warming temperatures [6] was distributed in the north-central regions and high mountain regions. Changes in the permafrost thermal regime can have certain impacts on local hydrology. Active-layer thickness and mean annual ground temperature at depths of 10–15 m ascended but geothermal gradient (°C m^-1^) at 15–50 m depth decreased in Mongolia during 2000s [34]. Hence, together with the increased precipitation, the enhanced permafrost thawing during 2000s may explain why many lakes in the north-central region showed a smaller reduction or increasing rates of lake area change. The above speculations require further analyses on regional hydrological processes including soil water change and regional atmospheric water vapor convergence (i.e. precipitation minus evapotraspiration) to describe the different hydrological regimes and varying waterways as well as their interrelations in Mongolia. The datasets necessary for the analyses are now partly available for the Northeast Asia from highly-resolved re-analysis climate datasets and satellite-based soil water content and evapotranspiration datasets [35, 36].

In arid and semi-arid regions in Mongolia, livelihood vulnerability of Mongolian nomadic herders is closely linked with regional drought-based disasters such as desertification, sand storm, and massive livestock kills [37, 38, 39]. Considering distinct seasonal nomadic movement of Mongolian herders [40], regional water-resource seasonality is of great concerns to determine success or failure of the nomadic system. Application of high temporal-resolution MODIS data enabled us to monitor multi-year seasonal variations of lake area. As results, the minimum lake area occurring in July or August showed 20% greater annual reduction rate (-52.0 km^2^ yr^-1^) than the maximum lake area (-42.5 km^2^ yr^-1^) for the 73 lakes investigated in this study. This implicates rising vulnerability of temporary lake water depletion during high summer season [41], which could give detrimental effects on local water demanding sectors such as livestock herding and vegetation growth in arid and semi-arid regions [38].

It is, however, unclear whether depletion or erratic reduction of lake water resources can be a useful indicator of the drought-induced disasters or livelihood vulnerability. For an instance, frequent dust storms occurred above normal in Mongolia in early 2000s [37]. Severe *dzud* disasters (i.e., a hydro-climatic winter disaster of massive livestock kills caused by harsh winter climate and/or summer drought conditions) happened twice in 2000s and killed about 30% of national livestock in Mongolia during 2000–2002 and 2009–2010 with harsh winter seasons following dry summers [38, 39]. Our results, however, showed that the lake area maintained above average during early 2000s indicating a failure of lake area as a national indicator of drought-induced disasters. This failure seems due to multiyear-scale time lags between precipitation and basin-level hydrological budget of some large-sized lakes that overwhelmingly determine the temporal pattern of national-scale lake area change. In contrast, a recent study showed that expansion or contraction of small or medium-sized lakes responds well to precedent precipitation at few-months temporal scale in Mongolia [25]. This implies better applicability of monitoring small-sized lakes for assessing the basin-scale drought occurrence and hence, the drought-induced disaster vulnerability. In a socio-hydrological point of view, this brings about again further refinement of lake area detection method compromising pros and cons of high-temporal but low-spatial resolution images that assures reliable monitoring of seasonal lake area for small or medium-sized lakes in arid and semi-arid regions.

## Supporting Information

S1 Fig A map of Fig 1 exported from ArcMap^™^ 10.0. (PDF)

S2 Fig A map of Fig 2 exported from ArcMap^™^ 10.0. (TIF)

S1 Table Datasets for Fig 3 including Landsat- and MODIS-derived lake areas (km^2^), perimeter-to-area ratio (km km^-2^), and relative error (%) of MODIS-derived lake area to Landsat-derived lake area. (XLSX)

S2 Table Datasets for Fig 4 including MODIS-derived lake areas (km^2^) for the seven lakes. (XLSX)

S3 Table Datasets for Fig 5 including timeseries of minimum, maximum, and mean lake areas (km^2^) derived from MODIS images. (XLSX)

S4 Table Datasets for Fig 6 including annual change rate of lake area (Slope, km^2^ yr^-1^) and level of significance (p_value). (XLSX)
