# Supplementary figures and images for: Assessing Seasonal and Inter-Annual Variations of Lake Surface Areas in Mongolia during 2000-2011 Using Minimum Composite MODIS NDVI
Source: PLoS One. 2016 Mar 23;11(3):e0151395. doi: 10.1371/journal.pone.0151395 (PMC4805288; doi:10.1371/journal.pone.0151395)

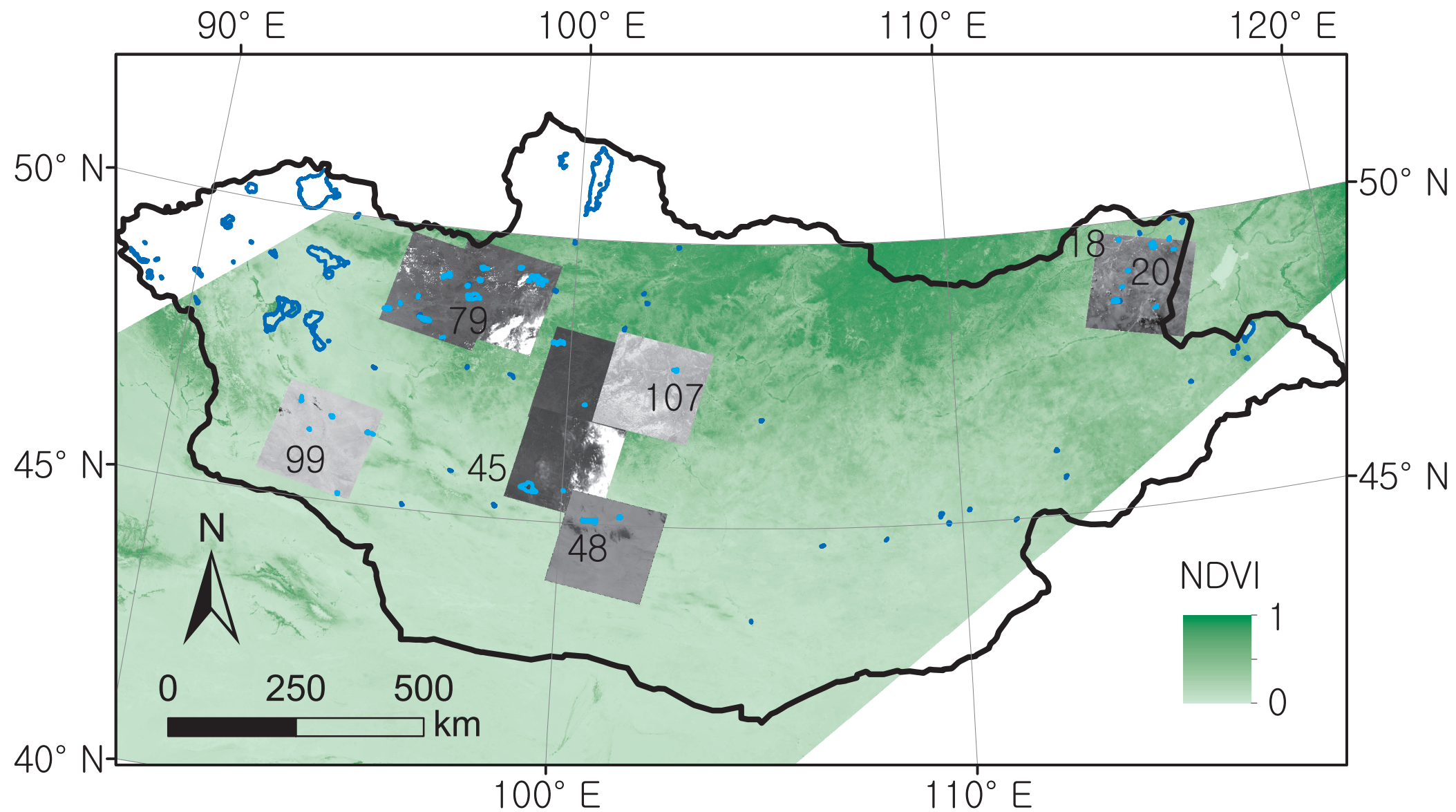

Supplement: S1 Fig — (PDF) [file pone.0151395.s001.pdf]
